# *In vivo *antimuscarinic actions of the third generation antihistaminergic agent, desloratadine

**DOI:** 10.1186/1471-2210-5-13

**Published:** 2005-08-18

**Authors:** G Howell, L West, C Jenkins, B Lineberry, D Yokum, R Rockhold

**Affiliations:** 1 Department of Pharmacology and Toxicology, University of Mississippi Medical Center, Jackson, MS 39216, USA; 2 Tougaloo College, Tougaloo, MS, USA

## Abstract

**Background:**

Muscarinic receptor mediated adverse effects, such as sedation and xerostomia, significantly hinder the therapeutic usefulness of first generation antihistamines. Therefore, second and third generation antihistamines which effectively antagonize the H_1 _receptor without significant affinity for muscarinic receptors have been developed. However, both *in vitro *and *in vivo *experimentation indicates that the third generation antihistamine, desloratadine, antagonizes muscarinic receptors. To fully examine the *in vivo *antimuscarinic efficacy of desloratadine, two murine and two rat models were utilized. The murine models sought to determine the efficacy of desloratadine to antagonize muscarinic agonist induced salivation, lacrimation, and tremor. Desloratadine's effect on the cardiovascular system was explored in both rodent models.

**Results:**

In the pithed rat, both desloratadine (1.0 mg/kg, i.v.) and the muscarinic M_2 _selective antagonist, methoctramine (0.5 mg/kg, i.v.), inhibited negative inotropic (left ventricular dP/dt) effects caused by oxotremorine, a nonselective muscarinic agonist (p < 0.05). Negative chronotropic effects caused by oxotremorine were inhibited by desloratadine, methoctramine, and the muscarinic M_3 _selective antagonist, 4-DAMP (1.0 mg/kg, i.v.). A late positive inotropic event observed after the initial decrease was inhibited by all three test compounds with desloratadine and 4-DAMP being the most efficacious. In the conscious animal, inhibition of baroreflex-mediated bradycardia was evaluated. Unlike atropine (0.5 mg/kg, i.v.), desloratadine did not alter this bradycardia. The antimuscarinic action of desloratadine on salivation, lacrimation, and tremor was also explored. In urethane-anesthetized (1.5 g/kg, i.p.) male ICR mice (25–35 g) desloratadine (1.0, 5.0 mg/kg) did not inhibit oxotremorine-induced (0.5 mg/kg, s.c.) salivation, unlike atropine (0.5 mg/kg) and 4-DAMP (1.0 mg/kg). In conscious mice, desloratadine failed to inhibit oxotremorine-induced (0.5 mg/kg, s.c.) salivation, lacrimation, and tremor. However, desloratadine did inhibit oxotremorine-induced tremor in phenylephrine pretreated animals.

**Conclusion:**

The presented data demonstrate that the third generation antihistamine, desloratadine, does not significantly antagonize peripheral muscarinic receptors mediating salivation and lacrimation, therefore, xerostomia and dry eyes should not be observed with therapeutic use of desloratadine. Our data also indicate when administered to a patient with a compromised blood-brain barrier, desloratadine may cause sedation. Patients with compromised cardiovascular systems should be closely monitored when administered desloratadine based on our results that desloratadine has the ability to interfere with normal cardiovascular function mediated by muscarinic receptors.

## Background

Antihistaminergic drugs are commonly classified into three generations. First generation antihistamines, such as diphenhydramine, effectively block the H_1 _receptor subtype but their use is limited due to significant central (sedation) and peripheral (tachycardia, xerostomia) antimuscarinic side effects. Second generation antihistamines, such as loratadine, retain a high selectivity for the H_1 _receptor and have fewer centrally mediated side effects than the first generation compounds because second generation compounds do not readily enter the central nervous system (CNS) [1]. However, two second generation antihistamines, astemizole and terfenadine, cause prolongation of the QT interval resulting in *torsades de pointes*. This adverse effect prompted the removal of terfenadine from the drug market [2]. The most recent, third generation compounds, include fexofenadine and desloratadine. These compounds are active metabolites of the second generation antihistamines, terfenadine and loratadine, respectively, and generally retain or surpass the H_1 _receptor selectivity of their parent compounds. For instance, desloratadine displays a higher affinity for the H_1 _receptor than does loratadine and antagonizes the human H_1 _receptor in a pseudoirreversible manner [3,4].

Questions remain concerning the potential for antimuscarinic adverse effects with desloratadine since both *in vitro *and *in vivo *experimentation indicates that desloratadine has the ability to block muscarinic receptors. Desloratadine demonstrated *in vitro *IC_50 _values of 48 nM and 125 nM against cloned human M_1 _and M_3 _muscarinic receptor subtypes, respectively [4]. *In vivo *muscarinic receptor blockade has been demonstrated in that desloratadine has been shown to inhibit pilocarpine induced salivation in mice and inhibit contractions of isolated rabbit and guinea pig iris smooth muscle [5,6]. Therefore, these data present the need to more definitively ascertain the potential antimuscarinic activity of desloratadine, *in vivo*. In the present study, several *in vivo *models were used to further assess antimuscarinic activity of desloratadine as well as the potential for penetration of the blood-brain barrier.

## Results

### Oxotremorine-induced tremor

**Figure 1:**
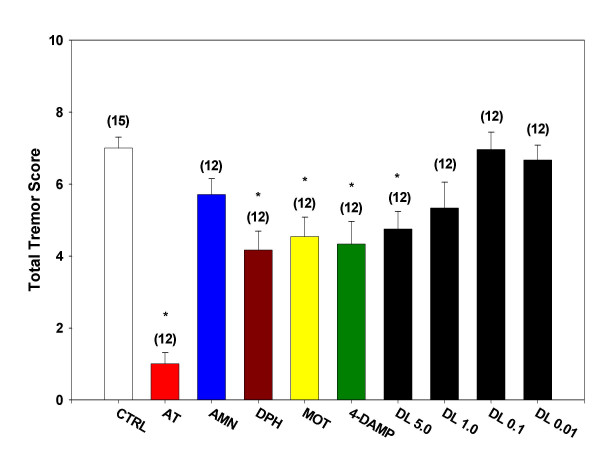
**Inhibition of oxotremorine-induced tremors. **Mice were treated with a single i.p. injection of one of the test agents (atropine sulfate, AT; atropine methyl nitrate, AMN; diphenhydramine, DPH; methoctramine, MOT; 1,1-dimethyl-4-diphenylacetoxypiperidinium iodide, 4-DAMP; desloratadine, DL) and placed in individual shoebox cages for observation. Fifteen minutes later, each mouse received a single s.c. injection of oxotremorine sesquifumarate (0.5 mg/kg) at the nape of the neck. At 5, 10 and 15 minutes following the oxotremorine injection, mice were observed for severity of tremor and for the presence of salivation and lacrimation. The sum of the scores for the three time points for tremor is presented as Total Tremor Score. Numbers in parenthesis represent the number of animals in each group and asterisk denotes statistical significance (P < 0.05) vs. control.

Intraperitoneal injection of oxotremorine (0.5 mg/kg) induced tremor in conscious mice. The only dose of desloratadine causing inhibition of oxotremorine-induced tremor was 5.0 mg/kg (Figure 1). Desloratadine (1.0, 0.1, and 0.01 mg/kg) did not significantly inhibit generation of tremor. Unlike atropine sulfate (0.5 mg/kg), atropine methyl nitrate (0.5 mg/kg) did not inhibit tremors which confirms the central locus for oxotremorine-induced tremors. Diphenhydramine (1.0 mg/kg) significantly inhibited the generation of tremor by oxotremorine as did administration of both 4-DAMP (1.0 mg/kg) and methoctramine (0.5 mg/kg) prior to administration of oxotremorine.

### Oxotremorine-induced tremor with phenylephrine pretreatment

**Figure 2:**
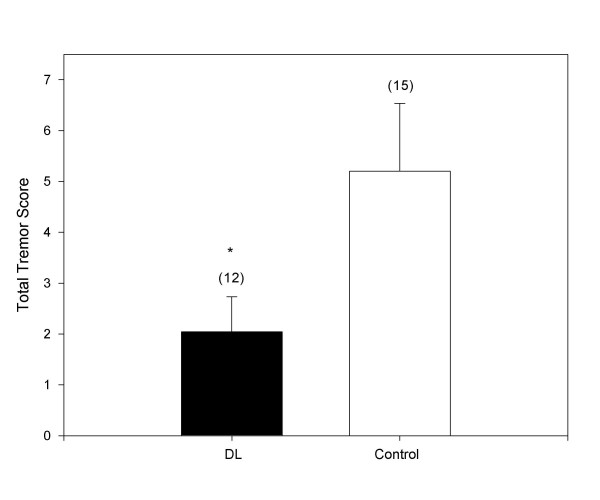
**Inhibition of oxotremorine-induced tremors with phenylephrine pretreatment. **Male ICR mice (30–35 g) were treated with a single i.p. injection of phenylephrine hydrochloride (10 mg/kg; 10 μL/g). Each mouse was then placed in an individual shoebox cage for observation. Five minutes after this, each mouse was given a second i.p. injection of either vehicle (control; 10 μL/g) or desloratadine (free base) at a dose of 1.0 mg/kg (DL). Fifteen minutes later, each mouse received a single s.c. injection of oxotremorine sesquifumarate (0.5 mg/kg) at the nape of the neck. At 5, 10 and 15 minutes following the oxotremorine injection, mice were observed for severity of tremor and for the presence of salivation and lacrimation. The sum of the scores for the three time points is presented as Total Tremor Score. Numbers in parenthesis represent the number of animals in each group and asterisk denotes statistical significance (P < 0.05) vs. control.

Pretreatment with the vasopressor agent, phenylephrine (10 μg/kg; PE), functions to disrupt the blood-brain barrier by inducing acute hypertension [7,8]. Blood-brain barrier disruption resulted in significant inhibition of oxotremorine-induced tremors by desloratadine (1.0 mg/kg) compared to the 1% DMSO vehicle as reflected by their respective total tremor scores (Figure 2). Pretreatment with PE followed by administration of desloratadine (1.0 mg/kg; i.p.) elicited an oxotremorine-induced total tremor score of 2.0 ± 0.7 (mean ± SD) whereas PE pretreatment followed by vehicle elicited an oxotremorine-induced total tremor score of 5.2 ± 1.3 (mean ± SD).

### Oxotremorine-induced salivation and lacrimation

**Figure 3:**
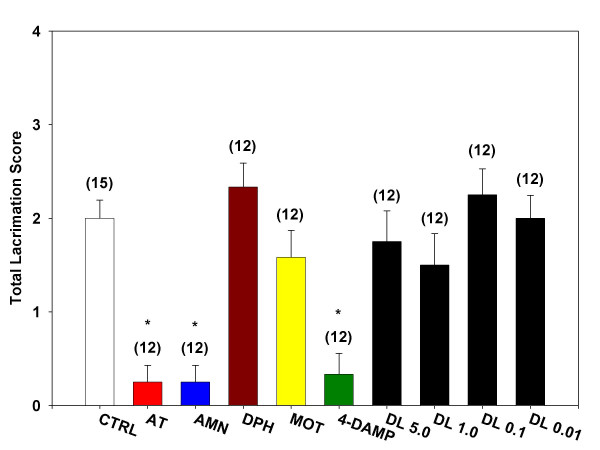
**Inhibition of lacrimation during oxotremorine-induced tremors. **Mice were treated with a single i.p. injection of one of the test agents (atropine sulfate, AT; atropine methyl nitrate, AMN; diphenhydramine, DPH; methoctramine, MOT; 1,1-dimethyl-4-diphenylacetoxypiperidinium iodide, 4-DAMP; desloratadine, DL) and placed in individual shoebox cages for observation. Fifteen minutes later, each mouse received a single s.c. injection of oxotremorine sesquifumarate (0.5 mg/kg) at the nape of the neck. At 5, 10 and 15 minutes following the oxotremorine injection, mice were observed for severity of tremor and for the presence of salivation and lacrimation. The sum of the scores for the three time points for lacrimation is presented as Total Lacrimation Score. Numbers in parenthesis represent the number of animals in each group and asterisk denotes statistical significance (P < 0.05) vs. control.

Administration of oxotremorine (0.5 mg/kg, i.p.) elicited salivation in conscious and urethane-anesthetized mice. In conscious mice, oxotremorine-induced salivation was not significantly inhibited by pretreatment with desloratadine (0.01, 0.1, 1.0, and 5.0 mg/kg) (Figure 3). However, salivation was significantly inhibited by pretreatment with atropine (0.5 mg/kg), atropine methyl nitrate (0.5 mg/kg), and 4-DAMP (1.0 mg/kg). Pretreatment with methoctramine (0.5 mg/kg) and diphenhydramine (1.0 mg/kg) failed to inhibit oxotremorine-induced salivation.

**Figure 4:**
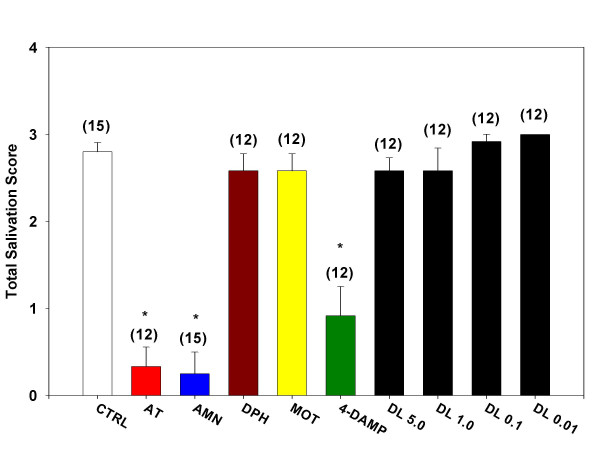
**Inhibition of salivation during oxotremorine-induced tremors. **Mice were treated with a single i.p. injection of one of the test agents (atropine sulfate, AT; atropine methyl nitrate, AMN; diphenhydramine, DPH; methoctramine, MOT; 1, 1-dimethyl-4-diphenylacetoxypiperidinium iodide, 4-DAMP; desloratadine, DL) and placed in individual shoebox cages for observation. Fifteen minutes later, each mouse received a single s.c. injection of oxotremorine sesquifumarate (0.5 mg/kg) at the nape of the neck. At 5, 10 and 15 minutes following the oxotremorine injection, mice were observed for severity of tremor and for the presence of salivation and lacrimation. The sum of the scores for the three time points for salivation is presented as Total Salivation Score. Numbers in parenthesis represent the number of animals in each group and asterisk denotes statistical significance (P < 0.05) vs. control.

Inhibition of oxotremorine-induced salivation in urethane-anesthetized mice yielded results similar to those obtained in conscious male ICR mice. Pretreatment with desloratadine (1.0 mg/kg) failed to significantly inhibit oxotremorine-induced salivation (Figure 4). As in the conscious animal, pretreatment with atropine (0.5 mg/kg) and 4-DAMP (1.0 mg/kg) significantly inhibited oxotremorine-induced salivation. Also, administration of diphenhydramine (1.0 mg/kg) and methoctramine (0.5 mg/kg) failed to significantly inhibit saliva production.

**Figure 5:**
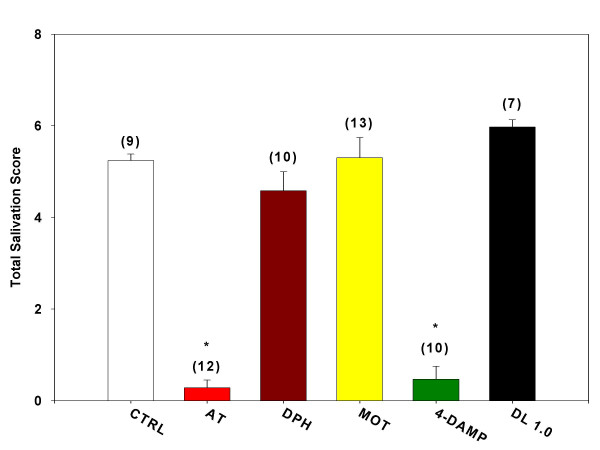
**Inhibition of oxotremorine-induced salivation. **Mice were anesthetized with urethane (1.5 g/kg, i.p.; 1 g/ml). Each mouse was then treated with a single i.p. injection of one of the test agents (atropine sulfate, AT; diphenhydramine, DPH; methoctramine, MOT; 1,1-dimethyl-4-diphenylacetoxypiperidinium iodide, 4-DAMP; desloratadine, DL). Each mouse was then placed prone and head-down on a plexiglass plate inclined at 10° and covered with a sheet of Whatman no. 3 MM filter paper. Fifteen minutes after administration of the test agent, a 0.5 mg/kg dose of oxotremorine was administered in a volume of 1 μl/g. Every five minutes for 30 minutes, each mouse was moved up the incline. Salivation production was measured immediately following the move at the end of each collection period by measurement of the circumference of the moist area of filter paper immediately beneath each mouse's mouth. The sum of the 6 collection periods is listed as Total Salivation Score. Numbers in parenthesis represent the number of animals in each group and asterisk denotes statistical significance (P < 0.05) vs. control.

Desloratadine (0.01, 0.1, 1.0, and 5.0 mg/kg) pretreatment had no significant effect on oxotremorine-induced lacrimation in conscious mice (Figure 5). As with inhibition of salivation in the conscious animal, pretreatment with atropine (0.5 mg/kg) and 4-DAMP (1.0 mg/kg) significantly inhibited oxotremorine-induced lacrimation. Pretreatment with either methoctramine (0.5 mg/kg) or diphenhydramine (1.0 mg/kg) did not significantly inhibit oxotremorine-induced lacrimation.

### Oxotremorine-induced changes in left ventricular contractility

**Figure 6:**
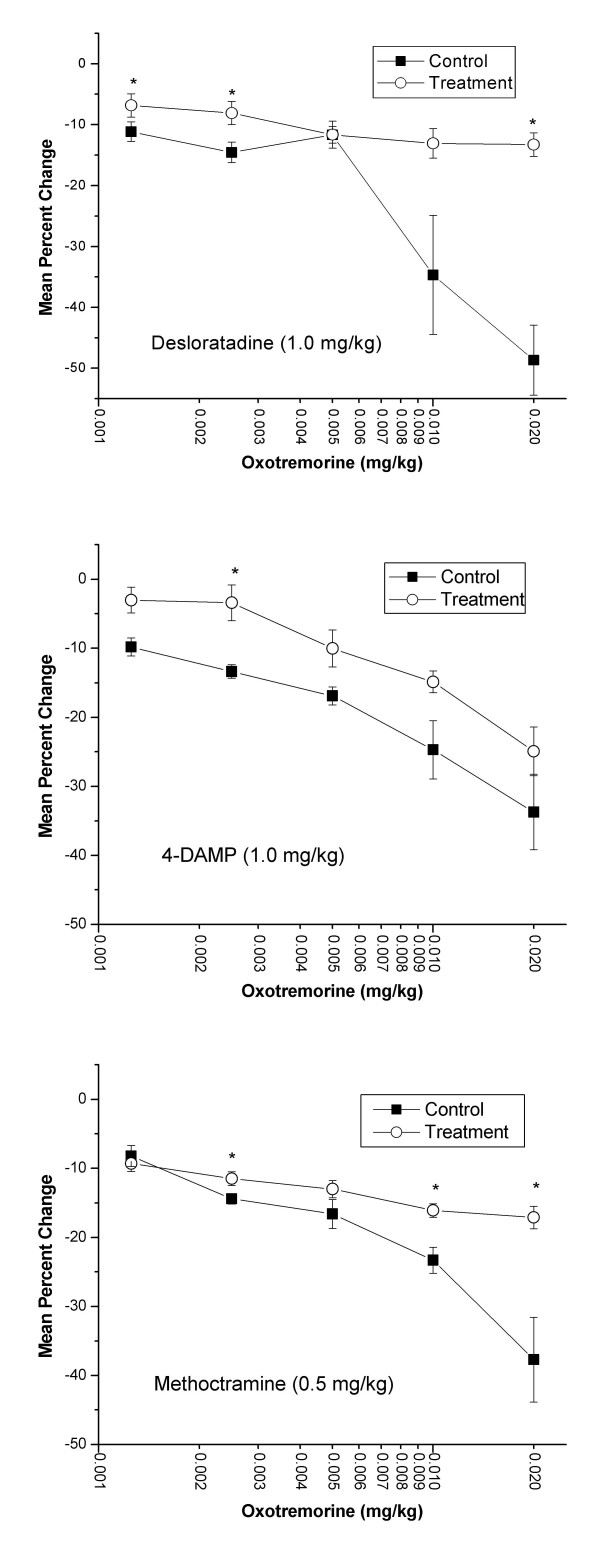
**Effect of desloratadine (DL), 4-DAMP, or methoctramine on oxotremorine-induced decrease in left ventricular contractility in the pithed rat. **Isoflurane-anesthetized animals (n = 6) were pithed after insertion of femoral and carotid arterial catheters. The carotid catheter was advanced into the LV to enable recording of contractility, which was expressed as the change in pressure over the change in time (dP/dt). Anesthesia was then discontinued. Following administration of atenolol (1.0 mg/kg, i.v.), oxotremorine was administered in random (n = 3) or ascending (n = 3) order of doses. The third generation antihistamine, DL (1.0 mg/kg, i.v.), the muscarinic M_3 _receptor antagonist, 4-DAMP (1.0 mg/kg, i.v.), or the muscarinic M_2 _receptor antagonist, methoctramine (0.5 mg/kg, i.v.), was then administered and the oxotremorine doses were repeated. No statistically significant differences were found between animals in which oxotremorine was given in random vs. ascending order of doses and both sets of data were pooled. Data are representative of the maximal percent fall in dP/dt compared to control following the administration of each dose of oxotremorine. The highest dose tested for oxotremorine (0.04 mg/kg, i.v.) could not be given prior to treatment with 4-DAMP. Statistical analysis was done using the paired t-test with P < 0.05 denoting a statistically significant difference versus control as indicated by an asterisk.

Intravenous injections of oxotremorine (0.00125-0.02 mg/kg) elicited biphasic inotropic responses. The initial phase consisted of a dose-dependent decrease in dP/dt. This decrease in inotropy began approximately 30–60 seconds after the beginning of oxotremorine injection and lasted for approximately 60 seconds. Both desloratadine and methoctramine treatments effectively blocked the negative inotropic effect (Figure 6). Administration of desloratadine (1.0 mg/kg) significantly inhibited oxotremorine-induced (0.00125, 0.0025, and 0.02 mg/kg) decreases in dP/dt indicated by a shift in the dose-response curve to the right. Also, administration of methoctramine (0.5 mg/kg) significantly inhibited oxotremorine-induced (0.0025, 0.01, 0.02 mg/kg) decreases in dP/dt indicated by a shift in the dose-response curve to the right. After administration of test agents, an additional dose of oxotremorine (0.04 mg/kg) was administered causing percentage decreases in left ventricular (LV) contractility of -14.2 ± 2.7, -38.3 ± 7.6, and -20.7 ± 1.5 for desloratadine, 4-DAMP, and methoctramine treatments, respectively (data not shown). Treatment with 4-DAMP had little antagonistic effect on the negative inotropic response to oxotremorine.

**Figure 7:**
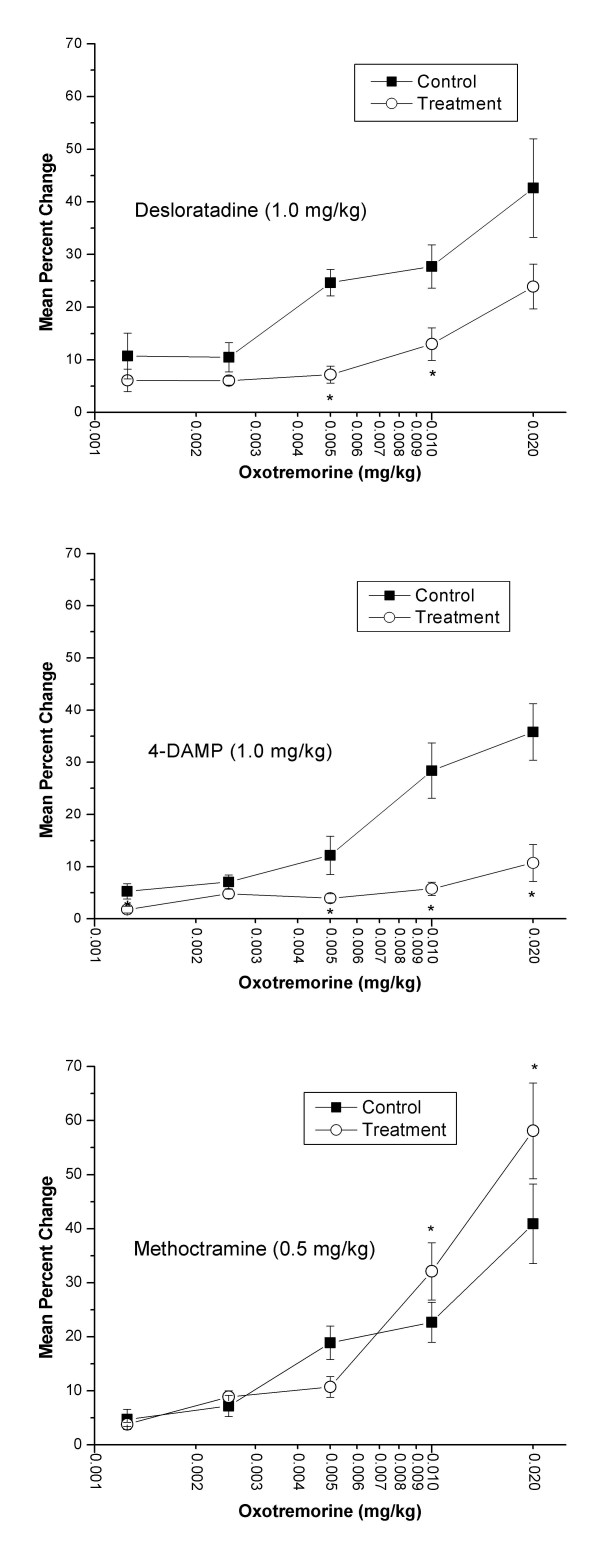
**Effect of desloratadine (DL), 4-DAMP, or methoctramine on oxotremorine-induced increase of left ventricular contractility in the pithed rat. **Isoflurane-anesthetized animals (n = 6) were pithed after insertion of femoral and carotid arterial catheters. The carotid catheter was advanced into the LV to enable recording of contractility, which was expressed as the change in pressure over the change in time (dP/dt). Anesthesia was then discontinued. Following administration of atenolol (1.0 mg/kg, i.v.), oxotremorine was administered in random (n = 3) and ascending (n = 3) order of doses. The third generation antihistamine, DL (1.0 mg/kg, i.v.), the muscarinic M_3 _receptor antagonist, 4-DAMP (1.0 mg/kg, i.v.), or the muscarinic M_2 _receptor antagonist, methoctramine (0.5 mg/kg, i.v.) was then administered and the oxotremorine doses were repeated. No statistically significant differences were found between animals in which oxotremorine was given in random vs. ascending order of doses and both sets of data were pooled. Data are representative of the maximal percent increase in left ventricular contractility compared to control following the administration of each dose of oxotremorine. The highest dose tested for oxotremorine (0.04 mg/kg, i.v.) could not be given prior to treatment with 4-DAMP. Statistical analysis was done using the paired t-test with P < 0.05 denoting a statistically significant difference versus control as indicated by an asterisk.

The second phase of the inotropic response to oxotremorine consisted of a dose-dependent increase in dP/dt. This increase immediately followed the initial decrease and had a duration of 2–5 minutes. Both desloratadine and 4-DAMP antagonized the oxotremorine-induced positive inotropic effect (Figure 7). Administration of desloratadine (1.0 mg/kg) significantly inhibited oxotremorine-induced (0.01 and 0.005 mg/kg) increases in dP/dt indicated by a shift in the dose-response curve to the right. Also, administration of 4-DAMP (1.0 mg/kg) significantly inhibited oxotremorine-induced (0.005, 0.01, and 0.02 mg/kg) increases in dP/dt indicated by a shift in the dose-response curve to the right. In contrast to desloratadine and 4-DAMP, methoctramine treatment (0.5 mg/kg) resulted in a statistically significant (P < 0.05) increases in dP/dt after oxotremorine (0.01 and 0.02 mg/kg) administration compared to control values recorded prior to methoctramine treatment. After administration of test agents, an additional dose of oxotremorine (0.04 mg/kg) was administered causing percentage increases in LV contractility of 32.2 ± 7.1, 20.5 ± 5.4, and 52.9 ± 9.5 for desloratadine, 4-DAMP, and methoctramine treatments, respectively (data not shown).

### Oxotremorine-induced bradycardia

**Figure 8:**
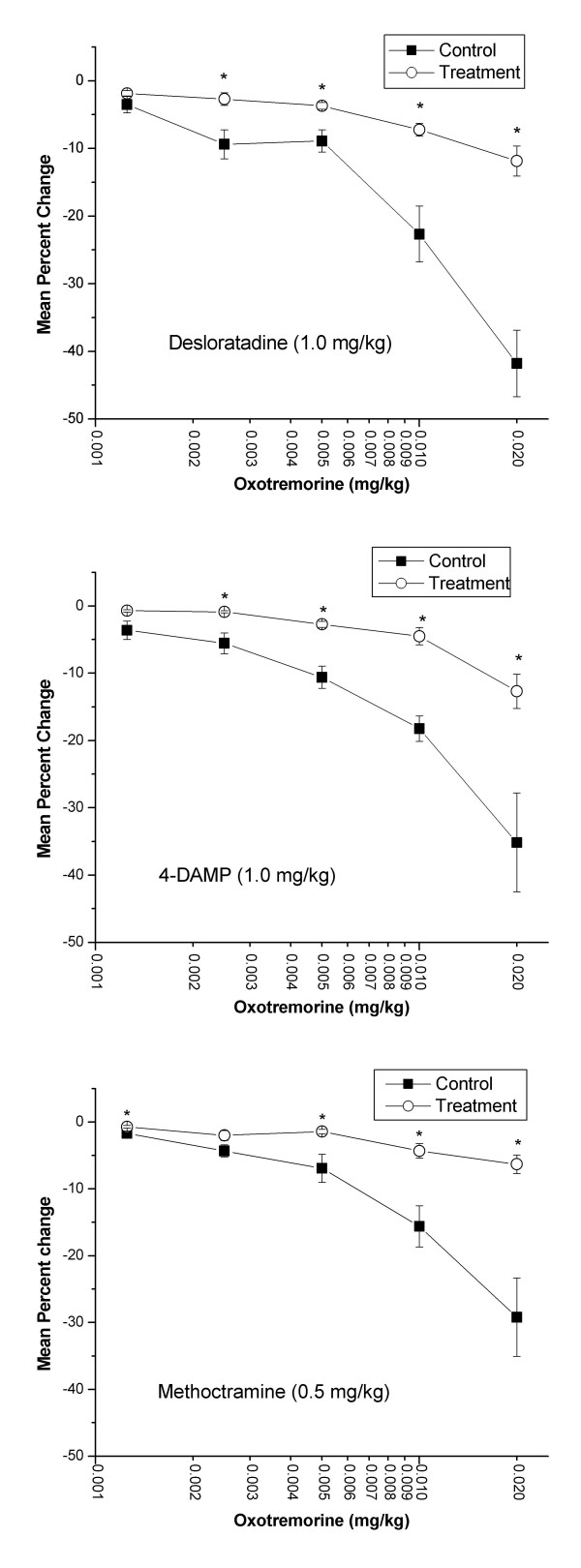
**Effect of desloratadine (DL), 4-DAMP, or methoctramine on oxotremorine-induced decrease of heart rate (HR) in the pithed rat. **Isoflurane-anesthetized animals (n = 6) were pithed after insertion of femoral and carotid arterial catheters. The femoral catheter was inserted approximately four centimeters into the femoral artery to enable recording of heart rate. Anesthesia was then discontinued. Following administration of atenolol (1.0 mg/kg, i.v.), oxotremorine was administered in random (n = 3) and ascending (n = 3) order of doses. The third generation antihistamine, DL (1.0 mg/kg, i.v.), the muscarinic M_3 _receptor antagonist, 4-DAMP (1.0 mg/kg, i.v.), or the muscarinic M_2 _receptor antagonist, methoctramine (0.5 mg/kg, i.v.), was then administered and the oxotremorine doses were repeated. No statistically significant differences were found between animals in which oxotremorine was given in random vs. ascending order of doses and both sets of data were pooled. Data are representative of the maximal percent fall in heart rate compared to control following the administration of each dose of oxotremorine. The highest dose tested for oxotremorine (0.04 mg/kg, i.v.) could not be given prior to treatment with 4-DAMP. Statistical analysis was done using the paired t-test with P < 0.05 denoting a statistically significant difference versus control as indicated by an asterisk.

Administration of oxotremorine caused a dose-dependent decrease in heart rate. All three of the test agents antagonized this decrease as indicated by a shift in the dose-response curve to the right (Figure 8). After administration of all three test agents, the negative chronotropic effects of oxotremorine (0.005, 0.01, and 0.02 mg/kg) were significantly inhibited. Treatment with desloratadine (1.0 mg/kg) or 4-DAMP (1.0 mg/kg) also significantly inhibited the negative chronotropic response to oxotremorine (0.0025 mg/kg) while methoctramine (0.5 mg/kg) treatment inhibited the response to oxotremorine (0.00125 mg/kg). After administration of test agents, an additional dose of oxotremorine (0.04 mg/kg) was administered causing percentage decreases in heart rate of -18.8 ± 3.5, -32.1 ± 7, and -19.7 ± 4.2 for desloratadine, 4-DAMP, and methoctramine treatments, respectively (data not shown).

### Inhibition of baroreceptor reflex

The ability of desloratadine to significantly alter the baroreceptor reflex was assessed in the conscious rat. Data were expressed as the percent change from corresponding control values of blood pressure and heart rate and subsequently analyzed by linear regression. The mean slope values were then analyzed for significant differences (data not shown). Administration of desloratadine (1.0 mg/kg) prior to stimulation of the baroreceptor reflex resulted in a slope value of -0.708 ± 0.03 (mean ± SE; n = 6) with the corresponding control slope value of -0.795 ± 0.03 (n = 6) which was not a statistically significant difference. Unlike desloratadine, administration of atropine (0.5 mg/kg) prior to stimulation of the baroreceptor reflex resulted in a slope value of -0.548 ± 0.03 (n = 6) with the corresponding control slope value of -0.670 ± 0.02 (n = 6) which was a statistically significant difference (P < 0.05).

## Discussion

The focus of the present experiments was to determine the degree of antimuscarinic effects exerted by desloratadine at M_2 _and M_3 _receptors, *in vivo*. The non-selective muscarinic receptor agonist, oxotremorine [9], was employed as the challenge agent in the murine and rat models. Relatively selective antagonists at the M_2 _and M_3 _receptors, methoctramine [10,11] and 4-DAMP [12,13], respectively, were used for comparison. Our results indicate the third generation antihistaminergic agent, desloratadine, possesses a significant degree of antimuscarinic activity, primarily against cardiac M_2 _and M_3 _receptor subtypes, using *in vivo *whole animal preparations. However, the doses at which these activities are demonstrated exceed those normally utilized for therapeutic antihistaminergic effects. In addition, while penetration of the blood-brain barrier by desloratadine is unlikely to occur at therapeutic doses [14], evidence has been obtained suggesting penetration can be achieved and result in significant central antimuscarinic effects if the blood-brain barrier is compromised by administration of a vasopressor agent.

Oxotremorine-induced tremor, salivation, and lacrimation in the mouse have been used by others to evaluate the presence of antimuscarinic actions of drugs of interest [13,15,16]. The elicitation of tremor by oxotremorine is centrally mediated [17,18] and blockade of this response gauges penetration of an antimuscarinic agent across the blood-brain barrier. Thus, blockade of oxotremorine-induced tremor is indirectly indicative of the potential for an antimuscarinic agent to exert central actions, such as sedation, following peripheral administration. In the presence of an intact blood-brain barrier, desloratadine did not exert significant blockade of oxotremorine-induced tremor, except at the highest dose tested (5.0 mg/kg) which caused roughly 30% reduction in tremor severity. In contrast, following treatment with the vasopressor agent, phenylephrine, to open the blood-brain barrier, a previously ineffective dose of desloratadine (1.0 mg/kg) caused a 60% reduction in tremor severity. These data suggest that while desloratadine is unlikely to exert central antimuscarinic effects at therapeutic dosages (5.0 mg recommended dose) in normal adults, considerably greater CNS penetration may occur when the blood-brain barrier is compromised. The significance of this when desloratadine is combined with a vasopressor decongestant or when infection may compromise the blood-brain barrier [19,20] remains for further study. The present results showing blockade by pretreatment with either methoctramine or 4-DAMP, indicate that oxotremorine-induced tremor is mediated by both M_2 _and M_3 _receptors in the mouse as has been previously demonstrated by others [13,21].

Both oxotremorine-induced lacrimation [22] and salivation [23] have been shown to be mediated selectively through the M_3 _receptor subtype, a mediation confirmed by the present study. Thus, while methoctramine pretreatment had no effect on either variable, 4-DAMP pretreatment was capable of reducing both lacrimation and salivation by 60–80% below control responses. In direct contrast, desloratadine inhibited neither lacrimation nor salivation at doses as high as 5 mg/kg.

The pithed, atenolol-treated rat provides a useful acute model with which to examine antimuscarinic drug action on the circulatory system in the absence of both basal and phasic sympathetic nervous system influences. The administration of oxotremorine, in this model elicits dose-dependent bradycardia, and biphasic effects on cardiac inotropy. Oxotremorine causes an initial decline in contractility, as determined by ventricular dP/dt, followed by a more prolonged positive inotropic phase. This biphasic inotropic response to a muscarinic agonist has been previously reported for acetylcholine, bethanechol, and carbachol in a variety of experimental species [24-27].

The rat heart contains multiple muscarinic receptors, including the M_1 _[28], M_2 _[21], and M_3 _[29] subtypes. Of these, the M_2 _subtype predominates based on reverse-transcriptase polymerase chain reaction (rt-PCR) data indicating the M_2 _subtype constitutes more than 90% of the total muscarinic receptor mRNA, therefore, supporting its role as the major mediator of muscarinic influence over the functional state of the myocardium [30]. However, Krejci and Tucek also demonstrated the presence of mRNA for M_1 _and M_3 _subtypes, each constituting less than 1% and 3%, respectively, of the total muscarinic receptor mRNA in the rat heart [30]. M_2 _receptor agonists elicit bradycardia and a negative inotropic response through inhibition of cardiac adenylyl cyclase and/or an increase in potassium conductance via the muscarinic potassium channel [31,32]. In contrast, effects mediated through the M_1 _and/or M_3 _receptors may lead to increased contractile strength, through enhanced activity of phospholipase C and subsequent downstream events leading to increased intracellular free calcium availability [29,33]. Wang *et al*. [34] have recently reviewed the existence of multiple muscarinic receptors in the mammalian myocardium and have emphasized the presence of and physiological functions exerted by M3 receptors. A lesser body of data supports functional actions of the M1 subtype.

Desloratadine, at a dose of 1.0 mg/kg, effectively antagonized bradycardia and both negative and positive inotropic responses elicited by oxotremorine. Assuming adequate selectivity between cardiac muscarinic receptor subtypes, our data suggest the ability of methoctramine to blunt oxotremorine-induced negative inotropic event and the ability of 4-DAMP to blunt oxotremorine-induced positive inotropic event to be indicative of M_2 _and M_3 _receptor mediation of these phenomena, respectively. In contrast, however, both methoctramine (0.5 mg/kg) and 4-DAMP (1.0 mg/kg) blunted oxotremorine-induced bradycardia. Therefore, the possibility exists that oxotremorine-induced bradycardia is mediated by both M_2 _and M_3 _receptor subtypes.

The context in which the present results are taken is worthy of discussion. Both *in vitro *receptor binding data [35-37] and results from prior *in vivo *studies [5,6,37] demonstrate a considerably greater affinity of desloratadine for histaminergic than muscarinic receptors (for reviews see, [38,39]). Desloratadine has been found to exhibit a peak plasma concentration of approximately 28 ng/ml in healthy volunteers following a therapeutic antihistaminic dose of its parent compound, loratadine [35]. Single oral doses of desloratadine of 5, 7.5, 10, and 20 mg yielded peak plasma concentrations of 2.18, 3.03, 3.80, and 8.08 ng/L in human volunteers [36]. In mice, desloratadine exhibits an ED_50 _of 0.15 mg/kg in reduction of histamine induced paw edema [37]. Cardelus *et al*. [5] noted local antimuscarinic effects following topical ocular administration of 1–10 mg/ml of desloratadine. However, it is unlikely that systemic concentrations of desloratadine would rise to levels approaching those in the present study following normal therapeutic dosages of desloratadine, a fact which has been emphasized by others [37]. Thus, the antimuscarinic actions of desloratadine demonstrated in the present study would, most probably, be of significance only in overdose situations.

## Conclusion

Our findings indicate that, at doses greater than those recommended for antihistaminergic therapy, desloratadine causes significant blockade of cardiac M_2 _and possibly cardiac M_3 _receptors, *in vivo*. This was demonstrated by significant inhibition of oxotremorine-mediated positive and negative inotropic events and bradycardia by desloratadine in the pithed rat. In contrast, desloratadine does not significantly antagonize the M_3 _receptor subtype responsible for salivation and lacrimation as demonstrated by the compound's inability to inhibit oxotremorine-mediated salivation and lacrimation in the conscious mouse and lacrimation in the anesthetized mouse. Also, under normal physiological conditions, desloratadine does not effectively cross the blood-brain barrier. However, upon disruption of this barrier, desloratadine has the potential for CNS penetration and muscarinic receptor blockade.

## Methods

### Drugs and solutions

Test agents included atropine sulfate, atropine methyl nitrate, diphenhydramine hydrochloride, methoctramine hydrochloride, 1,1-dimethyl-4-diphenylacetoxypiperidinium iodide (4-DAMP), and desloratadine. All were reconstituted in 1% DMSO / PBS, aliquoted into separate vials, and stored at -20°C until used. With the exception of desloratadine and 4-DAMP, all test agent concentrations were calculated using the salt weights. Atropine sulfate, atropine methyl nitrate, diphenhydramine hydrochloride, DMSO, oxotremorine sesquifumarate, atenolol, halothane, and urethane were all purchased from Sigma Chemical Co. (St. Louis, MO, USA). Other purchased agents were isoflurane (Abbott Laboratories; North Chicago, IL, USA), methoctramine hydrochloride (ICN Biochemicals, Inc.; Aurora, OH, USA), and 4-DAMP (Tocris; Ellisville, MO, USA). Desloratadine was provided by Aventis Pharmaceuticals (Bridgewater, NJ, USA).

### Animal experiments

Male Sprague Dawley rats (275–325 g) and male ICR mice (25–35 g) were purchased from Harlan Sprague Dawley and housed in plastic group shoebox cages in an AAALAC-approved Laboratory Animal Facility. Animals were housed under a twelve hour light-dark cycle with food and water *ad libitum*. Food was withheld twelve hours prior to experimentation or surgical procedures. All animal use protocols were approved by the University of Mississippi Medical Center Institutional Animal Care and Use Committee.

### Inhibition of oxotremorine-induced tremor, salivation, and lacrimation

A murine model was used to test the ability of desloratadine to antagonize muscarinic actions induced by administration of the muscarinic agonist, oxotremorine [15]. On the afternoon of an experiment, each mouse was weighed and placed in a clear shoebox cage for observation 15 minutes prior to any drug administration. Test agents for this experiment were vehicle, atropine sulfate (0.5 mg/kg), atropine methyl nitrate (0.5 mg/kg), diphenhydramine hydrochloride (1.0 mg/kg), methoctramine hydrochloride (0.5 mg/kg), 4-DAMP (1.0 mg/kg), and desloratadine (5.0, 1.0, 0.1, and 0.01 mg/kg). Each mouse was given a single intraperitoneal (i.p.) injection in a volume of 1 μl/g of one of the test agents. Fifteen minutes later, each mouse received a single subcutaneous (s.c.) injection of oxotremorine sesquifumarate (0.5 mg/kg), a non-selective muscarinic agonist, at the nape of the neck. At 5, 10, and 15 minutes following oxotremorine injection, each mouse was assessed for the degree of tremor and for the presence or absence of salivation and lacrimation. A modified five-point grading scale was used to evaluate tremor: 0 = no observable tremor; 0.5 = limb tremor observable when mouse is held by the tail with all feet off the cage bottom for 15 seconds; 1 = intermittent tremor, with bouts lasting from 3–5 seconds; 2 = intermittent tremor, with bouts lasting more than 5 seconds; or continuous, fine tremor noticeable on tail and ears; 3 = severe, continuous, whole-body tremor. Salivation and lacrimation were separately evaluated on a two-point scale: 0 = no observable salivation/lacrimation; 1 = salivation/lacrimation present. All responses were assessed by each of two observers with no knowledge of the pretreatment given each mouse. The grade for each mouse reflects the sum of the three consecutive observations as either Total Tremor, Total Salivation, or Total Lacrimation Score.

### Inhibition of oxotremorine-induced salivation

A second paradigm, using mice (25–35 g), anesthetized with ethyl carbamate (urethane, 1.5 g/kg, i.p., 1 g/ml) was used to independently evaluate putative M_3 _receptor blockade inhibition of oxotremorine-induced salivation. This was modified after similar methods described for use in the rat by Lavy and Mulder [16]. All mice were denied access to food, but not to water, for 16 hours prior to anesthetization. Test agents for this experiment were vehicle, atropine sulfate (0.5 mg/kg), diphenhydramine hydrochloride (1.0 mg/kg), methoctramine hydrochloride (0.5 mg/kg), 4-DAMP (1.0 mg/kg; free base) and desloratadine (1.0 mg/kg; free base). A single i.p. injection of one of the test agents or vehicle was administered in a volume of 1.0 μl/g five minutes following injection of urethane. Each mouse was then placed prone and head-down on a plexiglass plate inclined at 10° and covered with a sheet of Whatman no. 3 MM filter paper. Fifteen minutes after test agent administration, a 0.5 mg/kg (i.p.) dose of oxotremorine was given in a volume of 1.0 μl/g body weight. Each mouse was moved up the incline every five minutes for thirty minutes. Saliva production was quantitated at the end of each five-minute collection period by measurement of the circumference of the moist area of filter paper immediately beneath each mouse's mouth. The sum of values from the six collection periods was recorded as Total Salivation Score (TSS).

### Inhibition of oxotremorine-induced tremor with phenylephrine pretreatment

Mice (30–35 g) were treated with a single i.p. injection of phenylephrine hydrochloride (10 μg/kg; 10 μL/g) to elevate systemic blood pressure and open the blood-brain barrier [7,8]. Each mouse was then placed in an individual shoebox cage for observation. Five minutes after this, each mouse was given a second i.p. injection of either vehicle or desloratadine (free base) at a dose of 1.0 mg/kg. Fifteen minutes later, each mouse received a single s.c. injection of oxotremorine (0.5 mg/kg) at the nape of the neck. At 5, 10, and 15 minutes post oxotremorine injection, mice were observed for severity of tremor. The sum of the scores for the three time points is presented as Total Tremor Score.

### Inhibition of oxotremorine-induced changes in cardiac function

The influence of oxotremorine over cardiac function in a pithed rat model was employed to evaluate muscarinic receptor antagonistic properties of desloratadine [15]. Male rats (275–325 g) were acutely anesthetized with 2–4% isoflurane in medical grade oxygen. Polyethylene arterial (PE-50) and venous (PE-10) catheters and a tracheal cannula (PE-240) were surgically implanted to permit monitoring of arterial blood pressure and chronotropy, i.v. drug administration, and maintenance of respiration by means of a Harvard rodent respirator, respectively. A catheter (PE-50) was passed via the right carotid artery into the left cardiac ventricle for measurement of left ventricular dP/dt as an index of inotropy. Responses were obtained using either a Grass Model 7P20G differentiator and recorded on a Grass Model 7D polygraph (Grass Instrument Co.; Quincy, MASS, USA) or with PowerLab/16 SP data acquisition system using Chart for Windows v4.0 recording software (ADInstruments; Colorado Springs, CO, USA). Each rat was then pithed by insertion of a blunt stainless steel rod, 2 millimeters in diameter, through the orbit of the eye and passed through the brain and spinal column, thus destroying the central nervous system (CNS) from forebrain to the terminus of the spinal cord. Atenolol (10 mg/kg, 1.0 ml/kg, i.v.) was administered to obviate peripheral catecholamine-induced increases in cardiac function. After a 15 minute stabilization period, doses of oxotremorine (0.00125, 0.0025, 0.005, 0.01, 0.02, 0.1 mg/kg), were administered randomly and flushed with 0.1 ml of heparinized 0.9% saline. Subsequently, a single i.v. injection of one of three test agents was administered over a two minute period. The test agents were desloratadine (1.0 mg/kg, 1.0 ml/kg, i.v.), the selective M_3 _muscarinic receptor antagonist, 4-DAMP (1.0 mg/kg, 1.0 ml/kg, i.v.), or the selective M_2 _muscarinic receptor antagonist, methoctramine (0.5 mg/kg, 1.0 ml/kg, i.v.). All doses of oxotremorine were then repeated in the order they were given prior to administration of the test compound. Maximal changes in chronotropy and inotropy were measured with each injection. Values are expressed as percent of the control value taken immediately before injection of each dose of oxotremorine.

### Inhibition of baroreceptor reflex

Rats (300–325 g) were used to determine the ability of desloratadine to block the vagally-mediated bradycardic component of the baroreceptor reflex. Catheters were inserted into the femoral artery (PE-50) and femoral vein (PE-10) and exteriorized between the animal's shoulders. Animals were allowed to recover for a minimum of three days. The experiment lasted two days per animal. Before the baroreceptor reflex of each animal was measured, the animal was allowed an acclimation period. The first day consisted of control baroreceptor reflex measurement. On the second day, either desloratadine (1.0 mg/kg, i.v.) or atropine (0.5 mg/kg, i.v.) was given prior to baroreceptor challenge. After an acclimation period, baroreceptor reflex measurement was repeated. The baroreceptor reflex was initiated by increasing doses of both phenylephrine and sodium nitroprusside to increase or decrease blood pressure, respectively. Dosing was discontinued when a maximal change of 50 mmHg was achieved. Blood pressure was recorded with Powerlab Data Acquisition system via a transducer attached to the arterial line. The linear regression feature in Origin 6.0 (OriginLab Corp.; Northhampton, MA) analyzed data and the slopes compared with SigmaStat 2.0 (Jandel Scientific Software; San Rafael, CA).

### Statistical analysis

Data obtained from the murine models of oxotremorine-induced salivation, lacrimation, and tremor were analyzed using a one-way repeated measures ANOVA with a Dunn's *post hoc *test. Data obtained from animals pretreated with either phenylephrine or vehicle was analyzed via the paired t-test. Inhibition of oxotremorine-induced alterations in cardiac function before and after either desloratadine, methoctramine, or 4-DAMP administration was analyzed via the paired t-test with changes in cardiac function at each dose of oxotremorine being compared. In all statistical comparisons, P ≤ 0.05 was deemed statistically significant.

## Authors' contributions

G. Howell performed all of the *in vivo *cardiovascular testing, drug preparation, data analysis on cardiovascular results, and drafted the manuscript. L. West performed *in vivo *salivation, lacrimation, and tremor testing, drug preparation, and data analysis on salivation and lacrimation results. C. Jenkins, B. Lineberry, and D. Yokum assisted L. West with oxotremorine-induced tremor experimentation on conscious mice. R. Rockhold is the corresponding author and principal investigator.
